# Case Report: Trochlear Wedge Sulcoplasty, Tibial Tuberosity Transposition, and Lateral Imbrication for Correction of a Traumatic Patellar Luxation in a Miniature Companion Pig: A Case Report and Visual Description

**DOI:** 10.3389/fvets.2020.567886

**Published:** 2021-01-13

**Authors:** Jennifer Høy-Petersen, Joe S. Smith, Paul T. Merkatoris, Kelley E. Black, Cosette M. Faivre, Kristina G. Miles, Dane M. Tatarniuk, Karl H. Kraus

**Affiliations:** ^1^Faculty of Veterinary Medicine, The Norwegian University of Life Sciences, Oslo, Norway; ^2^College of Veterinary Medicine, Iowa State University, Ames, IA, United States; ^3^Biomedical Sciences, College of Veterinary Medicine, Iowa State University, Ames, IA, United States; ^4^Veterinary Diagnostic and Production Animal Medicine, College of Veterinary Medicine, Iowa State University, Ames, IA, United States; ^5^Veterinary Clinical Sciences, College of Veterinary Medicine, Iowa State University, Ames, IA, United States

**Keywords:** miniature pig, patellar luxation, tibial tuberosity transposition, sulcoplasty, medial patellar luxation

## Abstract

The objective of this case report was to describe successful surgical and post-operative management of a medial patellar luxation in a Vietnamese Potbellied Pig. A two-year old, castrated, Vietnamese Potbellied Pig presented to a veterinary teaching hospital for right pelvic limb lameness of 2 weeks duration. Upon physical examination a grade 3 patellar luxation was diagnosed on the right pelvic limb. Surgical repair included a trochlear wedge sulcoplasty, tibial tuberosity transposition, and lateral imbrication as described for canine patellar luxation. The pig was managed post-operatively with meloxicam and a physical therapy regimen of seven weeks duration. At recheck examination the pig was sound, no complications were observed, and the owners were satisfied with the outcome. As miniature companion pigs, such as Vietnamese Potbellied Pigs are currently increasing in popularity as pets, this case demonstrated that comparative techniques from other veterinary species should be considered when considering a treatment plan for a pig with a medial patellar luxation.

## Background

Miniature pigs are becoming more popular as animal companions. Exact figures for the amount of miniature pigs (*Sus scrofa domesticus*) that are kept as companion animals in the United States is limited, however it is estimated that there has been a drastic increase in their numbers over the past 17 years (1). Miniature pigs first started appearing as companion animals in North America in the late 1980's and since then their popularity has increased, with an estimated population of close to 1 million animals in North America (1). There are multiple breeds of miniature pigs (e.g., Vietnamese Pot Belly, Kunekune, Juliani. Yucatan, etc) which can live for an average of 15–20 years and weigh between 27 and 136 kgs. Due to their conformation, potbellied pigs are thought to be predisposed to pulled muscles, damaged ligaments and fractures of their back and limbs (1).

Patellar luxation is a commonly reported injury in small animal practice (2). In dogs, medial patellar luxation has been more commonly diagnosed than lateral patellar luxation (2). The injury is less commonly diagnosed in cats (3). In small breed dogs, traumatic patellar luxation can heal spontaneously, but more common surgical repair is necessary (4). Common surgical techniques utilized in small animals for repair of patellar luxation include soft tissue imbrication, soft tissue release, tibial tuberosity transposition, and femoral trochleoplasty (5–7).

Currently, the only reported orthopedic surgeries performed in potbellied pigs involve shoulder luxation repair using tension sutures, long bone fracture repair, femoral head ostectomy for the treatment of acetabular fracture and articular fracture management by elbow fixation (8–11). However, there are no published reports covering surgical techniques for treating medial patellar luxation in miniature pigs. In small animal practice, patellar luxation is a common orthopedic problem, especially in small canine breeds (12), and there are numerous reports of successful surgical management in dogs (12). There are also reports of surgery performed on food animals such as cattle (13), llamas (14), alpacas (15), and goats (16). The objective of this report was to describe a medial luxated patellar correction with tibial tuberosity transposition, and resultant rehabilitation therapy in a miniature companion pig.

## Case Presentation

### Clinical History

A 2-year-old, 22.6 kg castrated male Vietnamese Potbellied pig presented to the Food Animal and Camelid Hospital at Iowa State University's College of Veterinary Medicine for a 2 week history of “toe-touching” lameness. Two weeks prior, the pig was noted to have fallen while running across a wood floor and was immediately lame afterward. Other than recent lameness, the pig had an episode of suspected erythema multiformae that resolved without treatment 1 year prior.

#### Physical Examination

Examination was within normal limits with exception of lameness. On presentation, the right pelvic limb was noted as Grade 4/5 lame with occasional toe-touching, but no absolute weight-bearing observed. On palpation, the patella was permanently luxated and could be manipulated in a medial fashion back into the groove (Grade 3/4) (17). The pig was bright, alert and responsive, and had an appropriate body condition score (2/5). Radiographs taken by the referring veterinarian displayed no evidence of fracture.

#### Therapeutic Planning

**Figure 1:**
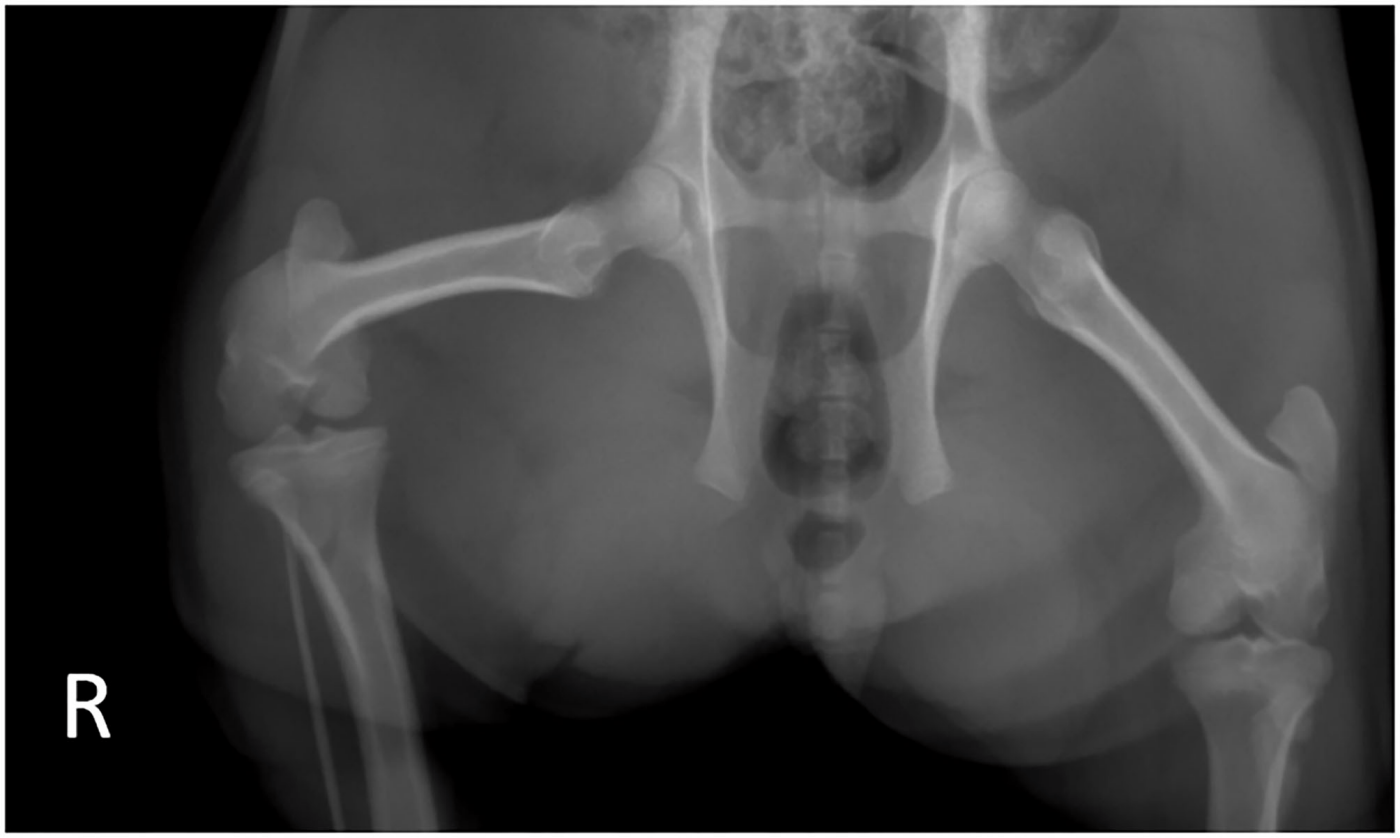
Preoperative radiographs taken by the referring veterinarian. Note the lack of apparent fractures in the right stifle.

Based on the confirmation of the patellar luxation and overall health of the patient, a surgical correction was planned for the next day. A single radiograph taken by the referring veterinarian showed no evidence of fracture or other orthopedic disease (Figure 1). The patient was withheld from food and water starting at midnight prior to the surgery.

#### Anesthesia

The patient was classified as ASA 2 according to the American Society of Anesthesiologists. Food was withheld for 18 h and water for 10 h, respectively, prior to anesthesia. Premedication included morphine at 0.4 mg/kg, dexmedetomidine at 0.005 mg/kg and midazolam at 0.5 mg/kg administered intramuscularly (IM). An intravenous catheter was placed in the right auricular vein and the patient was induced with propofol and ketamine, each at 1.1 mg/kg intravenously. The larynx was sprayed with 2% lidocaine prior to intubation with an 8 mm internal diameter cuffed endotracheal tube. Cardiovascular and respiratory monitoring was performed with a multiparametric monitor. Oxygen saturation using pulse oximetry, continuous electrocardiography, body temperature, oscillometric blood pressure, end-tidal carbon dioxide (EtCO_2_) using side-stream capnography, and gas analysis were measured; cardiovascular and respiratory parameters were recorded every 5 min. Anesthesia was maintained with isoflurane in oxygen delivered via a circle system. Mechanical intermittent positive pressure ventilation was instituted soon after intubation to maintain normocapnia (EtCO_2_ 35–45 mmHg) with a tidal volume of ~8 mL/kg and a peak inspiratory pressure of 16 cm H_2_O. A lumbosacral spinal epidural was performed using 0.15 mg/kg preservative free morphine and 2 mg/kg lidocaine for additional analgesia. The patient was placed in dorsal recumbency for the surgical procedure. Lactated Ringers Solution was administered at ~5 mL/kg/hr intraoperatively. Warming therapy included a forced-air warming blanket (Bair Hugger^TM^, 3M, Maplewood, MN, US) and warming blanket (HotDog®, Augustine Surgical Inc, Eden Prairie, MN, US) to minimize intraoperative hypothermia. Tulathromycin (Draxxin, Zoetis, Parsippany, NJ, US) at 2.5 mg/kg was administered IM shortly after induction for antimicrobial prophylaxis. Intraoperative complications included hypotension which was managed with fluid boluses, an anticholinergic, and a dopamine and dobutamine constant rate infusion at 10 mcg/kg/min. Total anesthetic time was 2 h and 15 min with surgical time being 63 min. The patient recovered unremarkably.

#### Surgical Procedure

**Figure 2:**
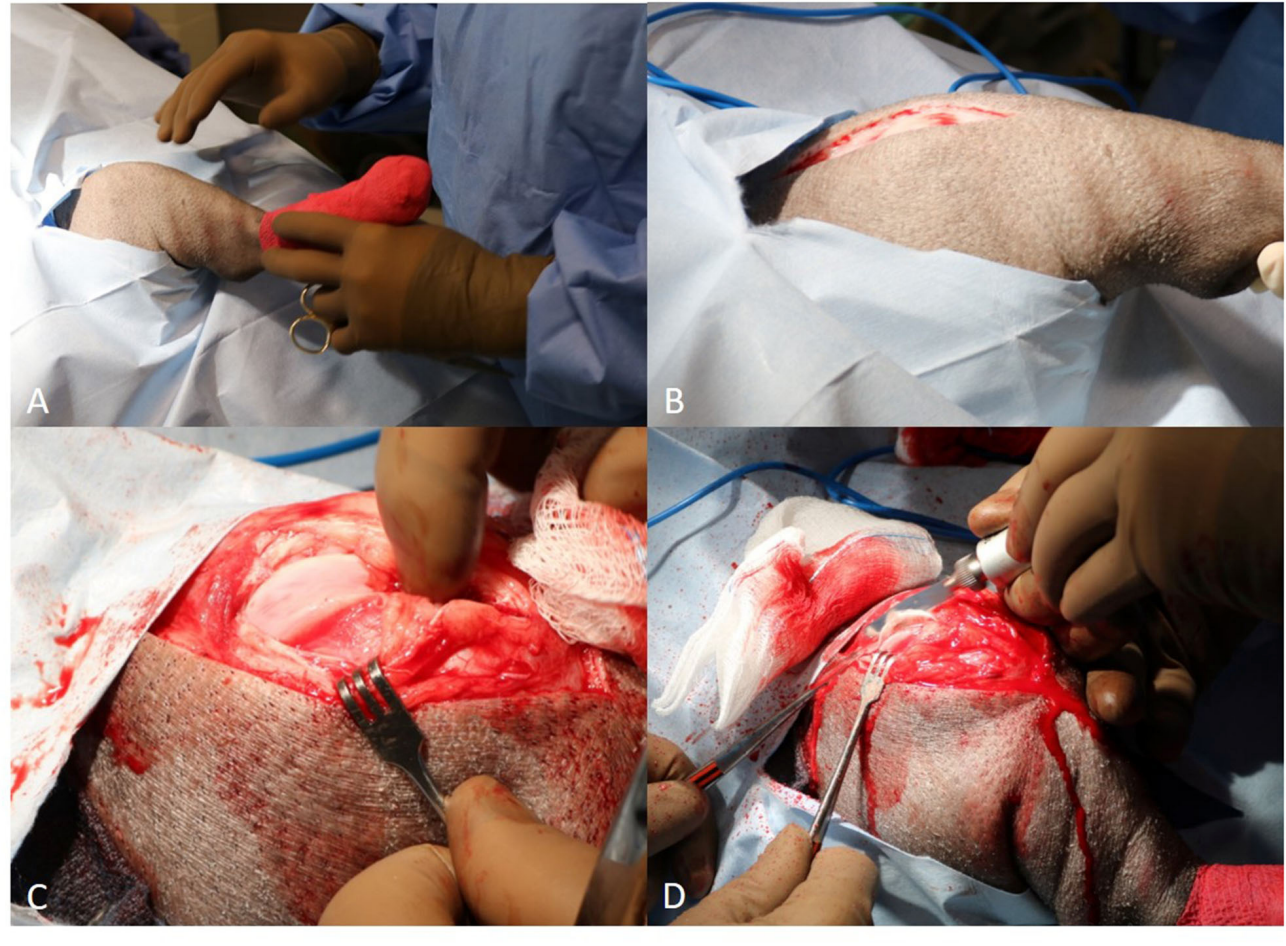
**(A)** Placement of the patient in dorsal recumbency with the patella pointing upwards (head is to the left of picture); **(B)** Craniolateral skin incision is created centered proximal-distal along the lateral border of the patella; **(C)** Manual luxation of the patella medially, which exteriorizes the trochlear groove. Note the shallow concavity present within the intertrochlear groove; **(D)** Trochlear wedge recession being performed with a reciprocating saw.

The patient was placed in dorsal recumbency. The right pelvic limb was clipped and aseptically prepared using the hanging leg technique. A #10 scalpel blade was used to make a 20 cm craniolateral skin incision on the stifle. The incision extended from proximal to the patella to the level of the proximal third of the tibia. Metzenbaum scissors were used to incise the subcutaneous tissue. A #15 blade was used to incise the retinacular fascia on the lateral aspect of the joint and a #15 blade was used to make a stab incision into the joint capsule. Curved Mayo scissors were used to extend the arthrotomy and the patella was luxated medially (Figure 2). Hemorrhage was controlled using radiopaque gauze sponges and electrocautery throughout the procedure. The joint was inspected. Cruciate ligaments were intact and meniscal cartilages were normal. The trochlear groove was extremely shallow. The articular cartilage appeared mildly hyperemic with several streaks of reddened foci apparent.

**Figure 3:**
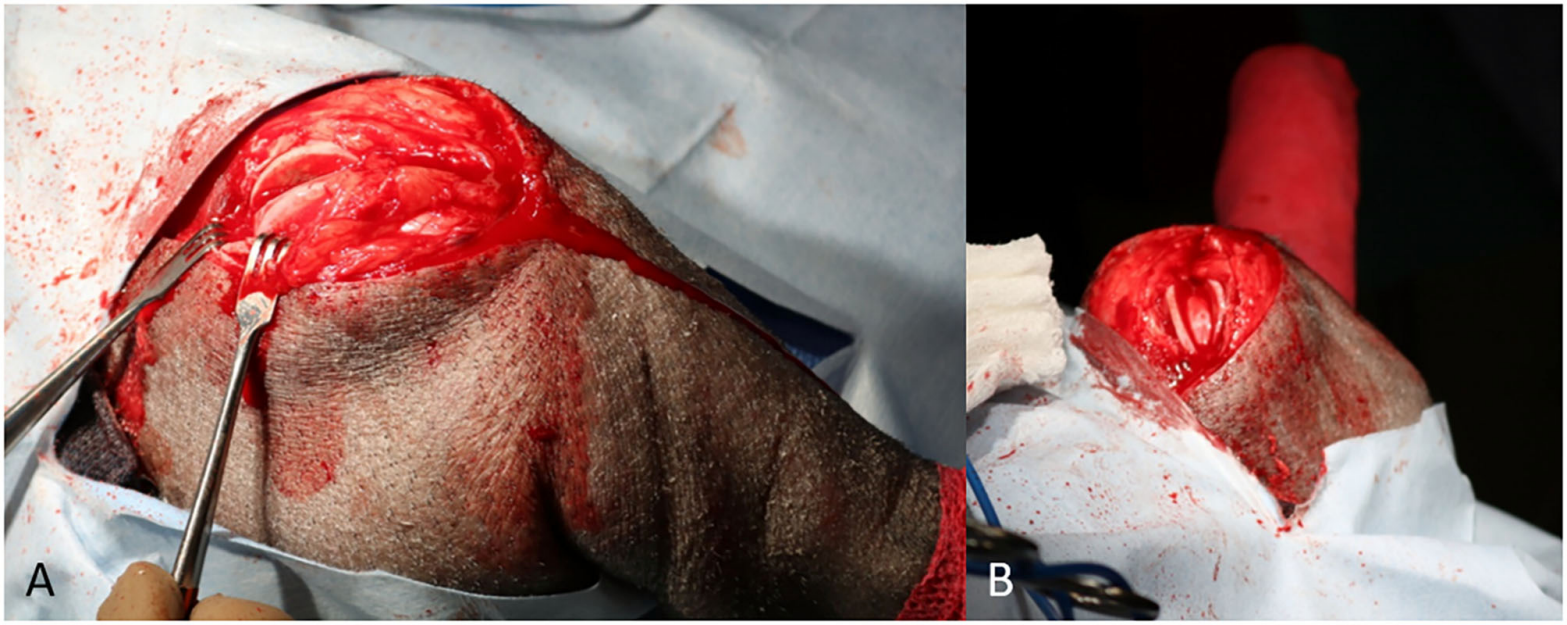
**(A)** Lateral to medial view of the trochlear groove following temporary removal of an osteochondral wedge segment; **(B)** Proximal to distal (skyline) view of the trochlear groove following recession and replacement of osteochondral wedge.

**Figure 4:**
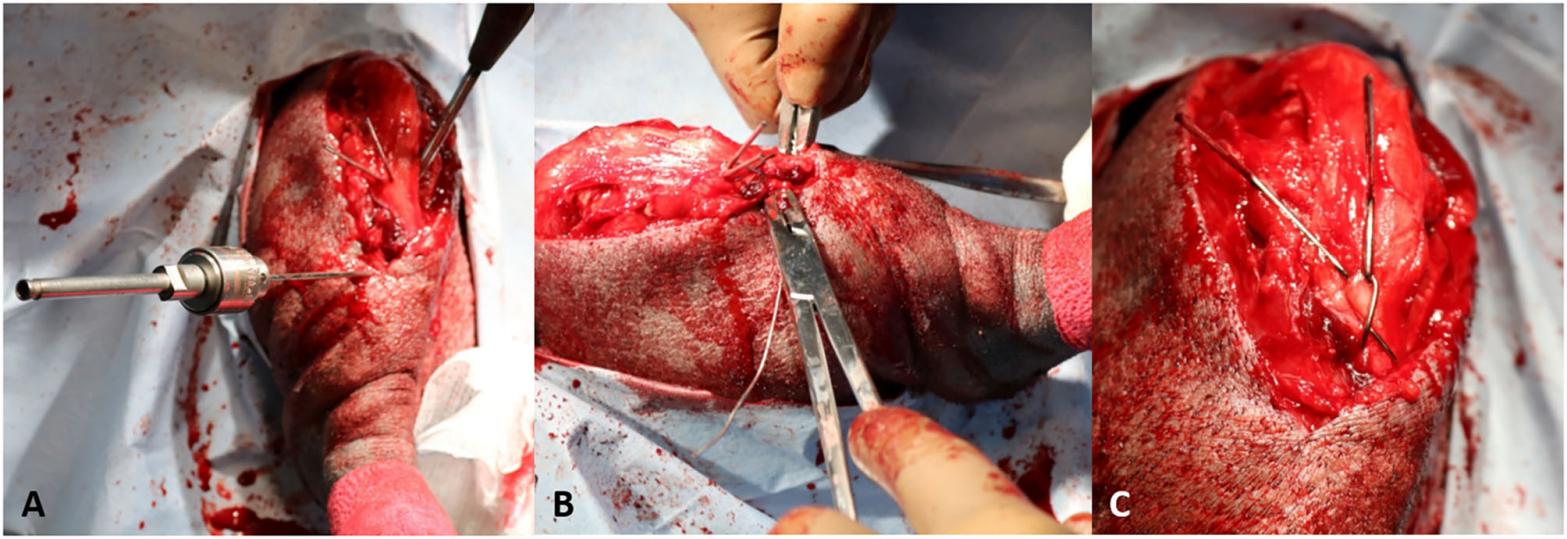
**(A)** The tibial tuberosity is transposed 0.75 cm laterally and secured with two 0.0625" Kirschner wires. A lateral to medial bone tunnel is manually drilled in the cranial cortex of the tibia immediately distal to the osteotomy; **(B)** After passing the wire through the distal bone tunnel, the wire was passed in a figure of 8 pattern to wrap over the proximally situated Kirschner wires; **(C)** The wires are tightened to secure the tension band. The Kirschner wires and cerclage wire are trimmed short.

**Figure 5:**
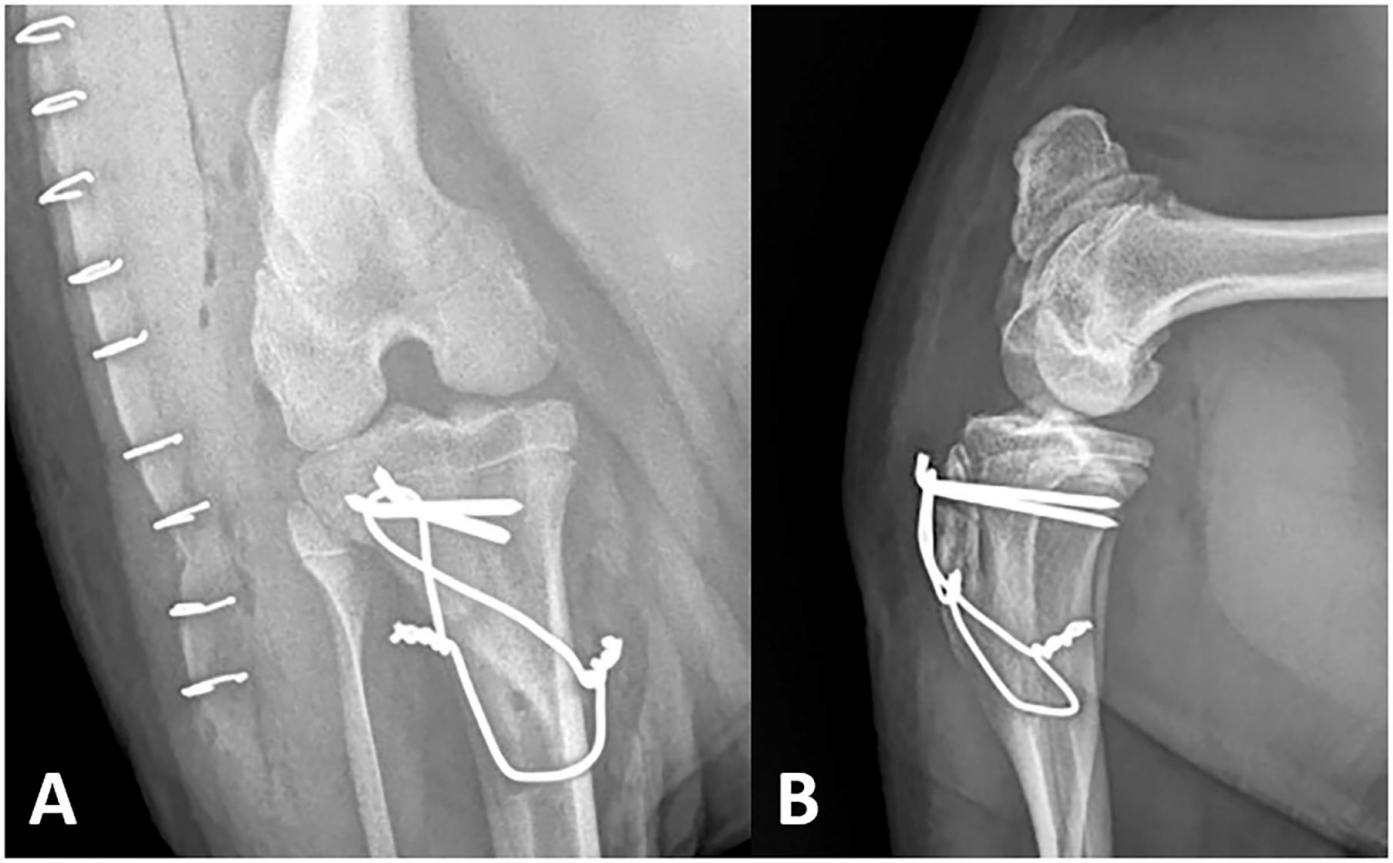
Post-operative right stifle. A vertical radiolucent tibial tuberosity transposition osteotomy is evident on the mediolateral **(A)** view. Stabilizing anchor pins, cerclage wire and bone tunnel are observed on both the craniocaudal **(B)** and mediolateral views. The position of the patella is normal.

A #15 blade was used to score the trochlear ridges of the femur for planned wedge recession. A reciprocating saw (Micro 100, Hall Surgical, Largo, Florida, US) was used to perform a wedge recession. Sufficient depth of the groove was confirmed by replacing the patella into the groove and afterwards ensuring a sufficient portion of the wedge was seated within the groove (Figure 3). The cranial tibial muscle was elevated from the lateral aspect of the tibial tuberosity and the fascia from the medial aspect of the proximal tibial was sharply reflected for planned tibial tuberosity transposition. The osteotomy was performed with the reciprocating saw. The tibial tuberosity was transposed ~0.75 cm laterally, a small K wire was placed. Two 0.0625 inch K wires were placed in the proximal tibia, and an 18 gauge tension band placed routinely (Figure 4). The stifle was reassessed for patellar luxation and no luxation in flexion or extension was noted. The joint capsule was closed with 0 polydioxanone (PDS, Ethicon inc, Somerville, NJ, US) using a horizontal mattress pattern. The lateral fascia was closed and imbricated with #0 polydioxanone in a mayo mattress pattern. The subcutaneous layer was closed with #2-0 poliglecaprone 25 suture (Monocryl, Ethicon inc) in a simple continuous pattern. The skin was closed with surgical staples. The incision was then covered with an aluminum aerosol bandage afterwards. Post-operative radiographs of the image were obtained (Figure 5).

#### Post-operative Care

Post-operative analgesia was administered by placing a fentanyl transdermal patch (50 mcg/kg/h) 7 h after the pre-anesthetic medications were administered. The next morning the patch was displaced by the patient. Repeated attempts to replace the patch were met with the patient removing the patches. Based on patient demeanor and attitude the patch was discontinued. Inflammation was managed postoperatively with meloxicam (0.5 mg/kg orally every 24 h).

During the first 24 h postoperative, the pig was confined to a 3 × 2 m stall divided in two, the one side bedded with hay. The other side was used to allow for the pig to defecate and urinate, as the patient was a house-trained companion animal. During recovery at the hospital the pig did not bear weight on his right pelvic limb, but was able to ambulate. The pig appeared to be otherwise doing well, not showing any obvious signs of pain or discomfort. Cryo-therapy (towel-wrapped ice pack applied for 15–20 min) was held against the incision every 6 h post-operatively. The pig was discharged 5 days post-operatively. Seven days post-operatively a second dose of tulathromycin (2.5 mg/kg, IM) was administered by the referring veterinarian. Meloxicam (generic, 0.5 mg/kg orally, q 24–48 h) was administered as needed, with the clients reporting no doses given after 3 weeks post-repair.

Initial physical restrictions involved putting the patient in a small room or crate when not being monitored as well as placing sections of carpet on the wood floor to minimize the risk of further slipping. The pig was also separated from other companion animals during this time. Physical rehabilitation therapy was performed during the first month postoperatively by focusing on massage of the limb (15 min intervals), immediately followed by 15 min of range of motion stretches. During stretching, care was taken to extend and flex the limb over a 15 s time period. The massage/stretching sessions were done three times daily for the first 4 weeks postoperatively. During this time, attempts were made to periodically and gently push the pig from the opposite rear limb in order to encourage bearing weight on the right pelvic limb. After 4 weeks the pig was reluctant to allow physical therapy for the full length of time previously described, and was also noted to be bearing more weight when walking. Approximately 5 weeks postoperatively, the pig was noted to spontaneously run around the house as he would before the incident.

**Figure 6:**
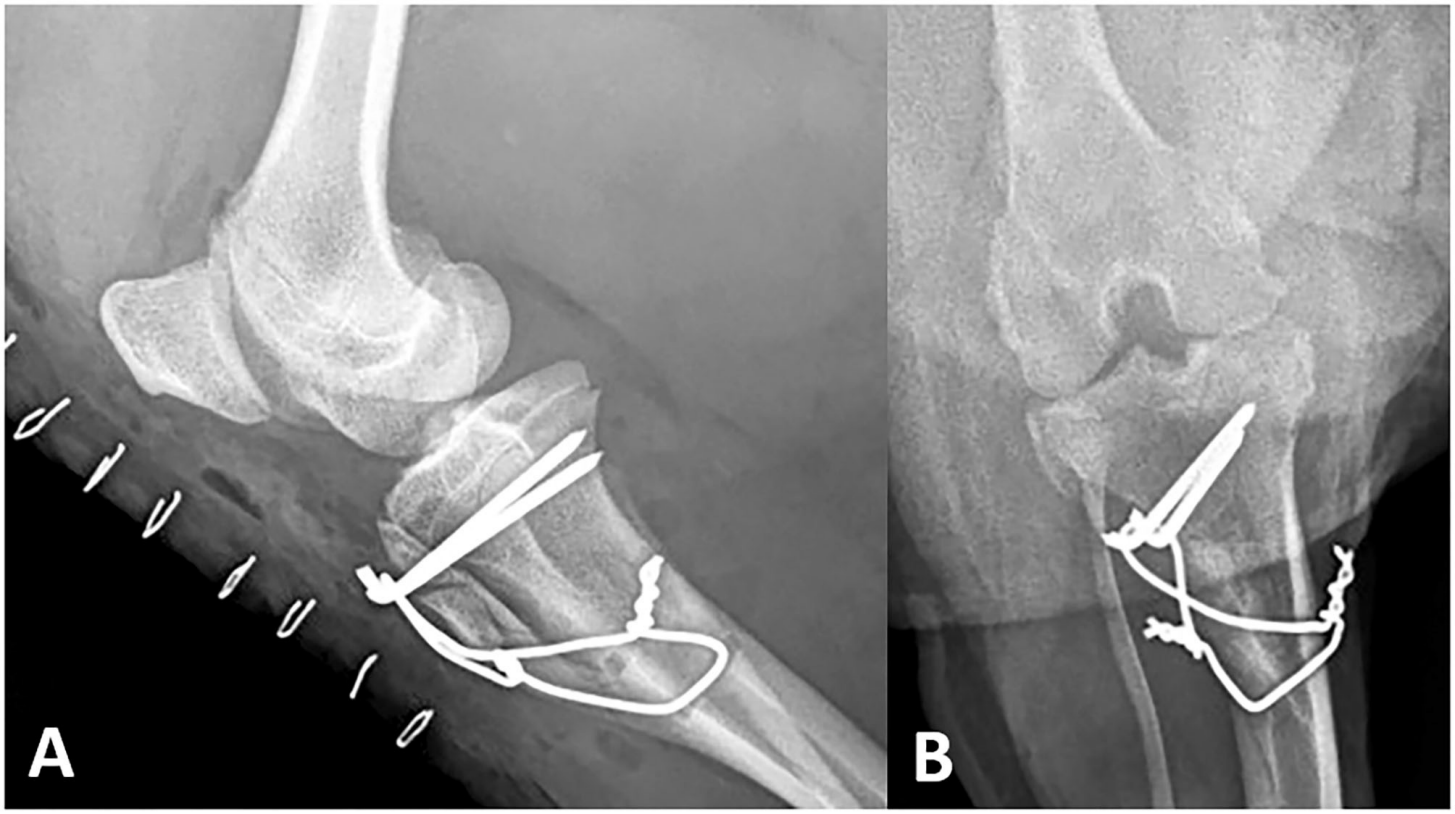
Seven week recheck right stifle. The distal half of the osteotomy exhibits a mineral opacity on the mediolateral view **(A)**. The position of the patella remains normal with no change in the appearance of the internal stabilization on the craniocaudal **(B)** or mediolateral views.

At recheck examination ~7 weeks post-surgery, the pig's vital parameters were within normal limits on physical examination. Occasionally, the pig would guard the right pelvic limb while standing, however no abnormal gait or posture was noted on ambulation. The clients reported at this time that the pig had resumed normal levels of activity compared to before the injury. Post-operative radiographs were obtained and considered normal (Figure 6).

## Discussion

This report presents the diagnosis, surgical treatment, post-operative management and physical rehabilitation of a companion miniature pig presenting to the Food Animal and Camelid Hospital of Iowa State University. A trochlear wedge resection and sulcoplasty combined with a tibial tuberosity transposition, as described for canine patients that sustain a similar injury, was performed. Physical rehabilitation relied upon range of motion exercises with progressively increasing movement over a seven week period. This report demonstrates a positive outcome based on the restoration of normal ambulation determined at recheck examination.

In dogs, medial patellar luxation is more common than lateral luxation (2, 12), and this may be similar to pigs based on this single case. Complications of patellar luxation repair in small animals include patellar reluxation, avulsion of the transposition site, or implant failure (18, 19). Recently, it has been suggested that in dogs there may be no difference in complication rate between techniques for medial patellar luxation repair (19). In dogs complications with this repair have been associated with body size, with higher complications observed in dogs >20 kg, than in dogs weighing <20 kg (5). Our case developed no significant complications post-operatively, suggesting that comparative techniques for dogs may be applicable to pigs. Caution should be used for grading other cases, as obesity can be prevalent in pet pigs, and a larger animal may not have as successful as an outcome.

Patellar luxations are not unique to small animals. In alpacas, conservative management of patellar luxations generally tend to result in unfavorable outcomes (20). However, spontaneous resolution has been reported in alpaca crias with patellar luxation (21). This may be an indication that not all patellar luxations require immediate surgical corrections. Nonetheless, there are multiple publications supporting the positive effects of surgical management in alpacas. The approaches described for this species involve imbrication (overlapping of layers of tissue to correct a defect) (22), trochlear wedge recession, tibial tuberosity transposition or a combination of these (15, 23, 24). One report demonstrated that such a combination of the mentioned methods resulted in an immediate improvement in weight bearing shortly after the procedure (15). These surgical techniques, with small adjustments, reflect the methods utilized in the present case and support their effectiveness in correcting this condition. Even though there are individual and species-based differences, these findings suggest that trochlear resection, tibial tuberosity transposition and imbrication appear to be appropriate and successful methods for correcting patellar luxations in alpacas and pigs.

Analgesia for this case consisted of meloxicam and fentanyl. NSAIDs such as meloxicam are recommended post patellar repair surgery in dogs (12) Meloxicam has ~87% bioavailability after oral administration in mature commercial pigs (25). The dose was initiated in this pig by the referring veterinarian (prior to presentation at the FACH), and was higher than the dose typically recommended for pigs of 0.3–0.4 mg/kg (26). With this in mind, and in order to minimize potential adverse effects, such as gastric ulceration, the clients were instructed to observe for bruxism and melena. If suspicion of ulceration was noted, a gastroprotectant, such as ranitidine or pantoprazole could be administered (9, 27), or meloxicam could be discontinued. Fentanyl has been used in both commercial and miniature pigs before (28, 29), although limited data exists for adverse reactions with this practice. In one case report of a pig in a research project that consumed a fentanyl patch, and exhibited dysphoria, panting and vocalization until depression was noted, that pig was successfully treated with naloxone (30). Other species, such as calves have reported adverse effects from fentanyl patch administration, including ataxia, bellowing, pyrexia, tachypnea, and tachycardia (31, 32), so clinicians should monitor patients with fentanyl patches for adverse effects. No adverse effects from fentanyl administration were noted in this case, however, this could be due to the short course of drug exposure from the patient removing the patch. Clinicians should consider patch security when applying them to future miniature companion pigs, as in other species ingestion of loose patches can have fatal outcomes (33, 34).

In previously reported patellar luxation cases in other companion or large animal species, there has been limited rehabilitation protocols reported, with cage confinement post patellar luxation repair most common. For non-tractable animals, cage confinement may be the sole option feasible and may be ample alone to ensure a positive outcome. This was supported in a case report documenting repair of patellar luxation in an Eurasian Lynx, where confinement to a small pen for 1 month was exclusively used, with no additional defined rehabilitation exercises (35).

The femur, patella and tibia of the stifle joint in the hind limbs of quadruped animals and is the equivalent of the human knee. A comparative study of the human knee to the stifle of six different animal species showed that the anterior cruciate ligament (ACL) has a variety of tibial insertion sites, depending on the species. The canine ACL tibial insertion was on the medial slope of the intercondylar eminence while the tibial insertion in the pig is separated by the anterior lateral meniscus, creating two distinct ACL bundles (36). This anatomical difference does not have a large impact on the choice of surgical techniques in the present case, as the cruciate ligaments were intact in the patient. In pigs, the patella is joined to the tibial tuberosity by a single patellar ligament as it is in carnivores and small ruminants, while three patellar ligaments (medial, intermediate or middle and lateral) are present in cattle and horse. Additionally in pigs, the lateral and medial trochlear ridges of the femur have the same height (as in humans and dogs), while in cattle and horse the medial trochlear ridge of the femur is higher. Because of this, traumatic medial luxation of the patella is extremely rare in cattle and horse. Another study also showed that the lateral meniscus is of larger dimensions than the medial meniscus (37), which also is the case for the dog (38). Our case report suggests that a comparative approach to patellar luxations can be utilized for the miniature pig, although more cases are needed.

Currently, there is no regulatory distinction between miniature companion pigs and pigs meant for human consumption in the United States. While not common, there are documented instances of miniature companion pigs entering the food chain (39). As such, clinicians need to select drugs and formulate therapeutic plans with prudent extra-label drug use in mind, as what would be impermissible for a production pig would be not permissible for a companion pig (9, 40–42). In this case the patient was administered meloxicam, tulathromycin, and fentanyl, as well as all anesthetic drugs in an extralabel fashion. To avoid angering the client the following message, as previously suggested (40): “While we understand that < name redacted> is a companion animal, because of his species we are legally obligated to inform you that his medications carry a withdraw time. Please contact us if you need this information.” In the author's experience, by utilizing this approach the communication regarding drug withdraw occurs, with less potential for angering the client by suggesting that their animal will be consumed.

Case reports provide value to the veterinary literature, but conclusions must be interpreted with the awareness that larger sample sizes will be needed. Future studies can help define whether other techniques for patellar luxation revision in companion miniature pigs may be suitable. For instance, partial parasagittal patellectomy, or polyethylene sulcal ridge prostheses may be applicable for companion pet pigs as well (3, 43). As our case was a smaller pig (24 kg) additional research is necessary to determine the effect of body size on outcome following patellar luxation repair in pet pigs, as size and breed predilections have been identified in canine patients (2, 5, 19).

### Conclusions

This report demonstrates that surgical correction via trochlear sulcoplasty, tibial tuberosity transposition, and imbrication can successfully be utilized to repair traumatic patellar luxation in companion pet pigs. Physical rehabilitation therapy, as similarly described for canine patients, is thought to help improve mobility and overall outcome, similar to canine patients. With the increasing popularity of miniature pigs as companion animals, veterinarians should consider these techniques as options when developing strategies for resolving patellar luxation in this specific species.

## Data Availability Statement

The original contributions generated in the study are included in the article/supplementary materials, further inquiries can be directed to the corresponding author.

## Ethics Statement

Ethical review and approval was not required for the animal study because this manuscript is a clinical case report conducted with written consent of the client. It is not a prospective research study. Written informed consent was obtained from the owners for the participation of their animals in this study.

## Author Contributions

JH-P and JS managed the clinical case, developed the surgical repair plan, and physical therapy plans as well as monitored patient follow up. PM, KM, DT, and KK developed the surgical repair plan. KB and CF contributed to case management and developed an anesthetic plan. All authors contributed to manuscript construction.

## Conflict of Interest

The authors declare that the research was conducted in the absence of any commercial or financial relationships that could be construed as a potential conflict of interest.
